# The Influence of Race and Gender on Receiving Assistance With Daily Activities Among Older Americans

**DOI:** 10.1093/geroni/igab060

**Published:** 2021-12-30

**Authors:** Chanee D Fabius, Lauren J Parker, Roland J Thorpe

**Affiliations:** Johns Hopkins Bloomberg University School of Public Health, Baltimore, Maryland, USA

**Keywords:** Disability, Disparities, Long-term services and supports

## Abstract

**Background and Objectives:**

Nearly 8.2 million community-dwelling, older Medicare beneficiaries receive support from long-term services and supports (LTSS) with routine daily activities. Prior work demonstrates disability-related disparities; however, it is unclear whether these patterns persist among LTSS recipients and across specific sets of activities. We examine race and gender differences in receiving help with self-care (e.g., eating), mobility (e.g., getting around the house), and household (e.g., shopping) activities in a nationally representative sample of community-dwelling Medicare beneficiaries receiving LTSS.

**Research Design and Methods:**

Cross-sectional analysis of 1,808 White and Black older adults receiving assistance with routine daily activities in the 2015 National Health and Aging Trends Study. Bivariate statistics were used to describe the sample and provide comparisons of characteristics by race and gender. Logistic regression models examined race and gender differences in receiving assistance with self-care, mobility, and household activities after adjusting for sociodemographic and health characteristics.

**Results:**

Race and gender differences were observed across all sociodemographic and health characteristics, as well as for all forms of assistance. Relative to White men, Black men had lower odds of receiving help with self-care activities. White and black women had higher odds and Black men had lower odds of getting help with mobility activities than White men. Black men and White and Black women all had higher odds of receiving assistance with household tasks compared to White men.

**Discussion and Implications:**

Our findings indicate that, despite prior evidence of disability-related disparities, the receipt of help with self-care, mobility, and household activities varies by race and gender. Findings reveal several target areas for future research. Future work should examine the role of cultural and social preferences for care, as well as the appropriateness of help, as evidenced by health service use and changes in quality of life.

**Translational Significance:** As a result of population aging and growing diversity, there is an increasing need to better understand the group of older adults who receive paid and unpaid LTSS in the community to help with routine daily activities. We find that the receipt of self-care, mobility, and household support varies by race and gender. Findings provide a basis for future analyses to examine preferences for and appropriateness of services for racially diverse men and women.

Nearly 8.2 million of all community-dwelling older Medicare beneficiaries receive an average of 36 h per week of long-term services and supports (LTSS) from family or unpaid or paid caregivers, including personal help with daily tasks such as self-care (e.g., eating, dressing), mobility (e.g., transferring in and out of bed, getting around the house), and household (e.g., shopping, meal preparation) activities (Freedman & Spillman, 2014; LaPlante et al., 2002). Receiving assistance with these tasks is associated with lower quality of life (Gobbens, 2018), hospitalizations (Na et al., 2017), and nursing home entry (Friedman et al., 2005). Most older adults prefer to receive assistance at home from unpaid family or friends or paid caregivers (Robison et al., 2014), although cultural preferences or societal expectations might influence the provision of care in home and community settings (Nkimbeng & Parker, 2021). For example, the provision of care may vary for women, who, as a result of societal expectations, are more often primary caregivers for partners and family members (American Association of Retired Persons, 2020; Sharma et al., 2016). Prior work has demonstrated that there are race and gender disparities in the prevalence of limitations with routine daily activities as older Black Americans and women experience higher rates of disability (Ciol et al., 2008; Murtagh & Hubert, 2004). However, it is unclear whether and how race and gender intersect to influence differences among those with the greatest need for LTSS, who are receiving assistance with daily activities (MACPAC, 2016). More specifically, little is known about how help varies by race and gender for specific groups of activities (i.e., self-care, mobility, and household activities). In the coming years, more older Americans will receive help with routine daily activities, thereby leading to an increasing demand for LTSS (Colby & Ortman, 2015; Thomas & Applebaum, 2015). Knowledge about whether the receipt of different forms of assistance varies across race and gender groups among LTSS recipients may help researchers, providers, and policymakers better understand differences that reflect variations in preferences for, access to, or availability of help. This is particularly important given that most frail and vulnerable older adults will rely on assistance from family, friends, and other community-based LTSS to help them as they age.

Self-care activities (i.e., bathing dressing, eating, toileting) and some mobility activities (i.e., getting around inside and outside of the home, getting in and out of bed) are most aligned with what are considered to be activities of daily living that are required for people to live on their own (Katz et al., 1963). Household activities include shopping, doing laundry, meal preparation, banking, and medication management, which are most closely related to instrumental activities of daily living (Lawton & Brody, 1969). These are different sets of activities—self-care and mobility activities are necessary for daily functioning, and household activities are critical to maintaining independence in the community as well as at home (Gifford et al., 2019). Prior work has demonstrated that receiving assistance with routine daily activities varies across several characteristics, such as age, gender, education, and Medicaid-enrollment status (Congressional Budget Office, 2013; Freedman & Spillman, 2014) and living arrangements (Henning-Smith et al., 2018). The availability of family and paid caregivers also likely contributes to the receipt of help (Spillman & Pezzin, 2000). Findings on race differences in use of paid help to assist with routine daily activities are mixed (Fabius et al., 2018, 2019; Feng et al., 2011). Factors contributing to disparities in use of paid help range from preferences for care to race and gender discrimination and medical mistrust, as is the case in other settings (e.g., primary care; Arnett et al., 2016; Fabius et al., 2018; SteelFisher et al., 2019).

Relative to older men, older women are more often living with more health conditions, functional limitations, and cognitive impairment and are overrepresented among those receiving Medicaid benefits due to low income and greater disability (The Henry J. Kaiser Family Foundation, 2013). While older women experience lower rates of mortality and use health care services more often than men, they more often receive assistance with daily activities (Freedman & Spillman, 2014; Thomas & Applebaum, 2015) and report greater disability than their male counterparts (Ciol et al., 2008; Dunlop et al., 2002). Additionally, compared to men, women have a longer duration of life lived with disability—this is partly due to higher prevalence of nonfatal chronic conditions (Vincent et al., 2010). However, despite their longer lives, women do not live more active years than men as a result of the increased prevalence of disability (Freedman et al., 2016), indicating that they may be more likely to experience adverse health outcomes and increased health care utilization.

Overall, Black older adults report poorer self-rated health, have lower education attainment, greater cognitive and mental impairment, and higher rates of hypertension, diabetes, and stroke that are associated with LTSS use (Fields et al., 2016) than their White counterparts. Black older adults also more often report receiving assistance with daily tasks, such as eating, dressing, or getting around inside or outside the house, and have a greater risk of developing mobility limitations (Coustasse et al., 2009; Freedman & Spillman, 2014; Nuru-Jeter et al., 2011; Vásquez et al., 2018). Additionally, Black men and women are more likely to experience a greater prevalence of disability compared to White older adults (Fuller-Thomson & Guralnik, 2010).

Research has demonstrated gender and race differences in the receipt of assistance with daily activities, but few studies have considered the intersection of both factors. Additionally, there is an increasing need to better understand the group of older adults who receive paid and unpaid LTSS in the community. Therefore, considering prior findings, and to address the current gap in the literature, we examine whether there are race and gender differences in receiving assistance with self-care, mobility, and household tasks among Black and White male and female Medicare beneficiaries getting help with daily activities.

## Method

This study used data from the 2015 round of the National Health and Aging Trends Study (NHATS). NHATS is an annual in-home survey that was designed to produce a nationally representative cohort of all Medicare enrollees aged 65 or older living in the contiguous United States. The survey oversamples persons in older age groups of Black non-Hispanic race and ethnicity (DeMatteis et al., 2016b). The first round of the survey occurred in 2011, with annual follow-ups. In 2015, the sample was replenished. The overall unweighted response rate for Round 5 was 76%, yielding a sample of 8,334 older adults.

Respondents were excluded if they self-identified as Hispanic, American Indian, Asian, Native Hawaiian, or were missing data on race/ethnicity (*n* = 921). We restricted the sample to exclude individuals living in nursing homes or residential care settings (*n* = 1,158). To identify those with the greatest need for LTSS, we restricted our sample to those receiving help with at least one self-care, mobility, or household task (for a health reason; MACPAC, 2016). This process yielded a final analytic sample of *N* = 1,808 Black and White older adults living in the community who were receiving assistance with daily activities.

### Assistance With Daily Activities

NHATS includes several measures reflecting activity limitations (Kasper & Freedman, 2019). The outcome variables (self-care, mobility, or household activities) were derived from questions that asked respondents whether they had received assistance with an activity in the last month. Respondents were considered to have received help if they reported that they received help from someone with a self-care (bathing, eating, dressing, and toileting), mobility (going outside of the home, getting around inside the home, and getting out of bed), or household task for a health reason (laundry, shopping meal preparation, medication administration, and banking) or could not complete the activity. We created three dichotomous variables indicating receiving help with at least one task in each category, as done in prior studies (Freedman & Spillman, 2014; Garfield et al., 2015).

### Independent Variables

Our primary independent variable of interest measures self-reported race and gender, coded as White men, White women, Black men, and Black women. Sociodemographic variables included age group (65–74, 75–84, and 85 and older), education (less than high school graduate, high school graduate/General Educational Development [GED], and more than a high school graduate), and self-reported Medicaid enrollment. Health characteristics include self-rated health and specific health conditions. Self-rated health responses included excellent, very good, good, fair, and poor. Due to small cell sizes for Black men in the excellent self-rated health category (*n* = 2), we collapsed the excellent and very good categories into one category. We also included specific health conditions associated with LTSS use, including arthritis, heart attack, heart disease, diabetes, stroke, and dementia (United States Commission on Long-Term Care, 2013). Dementia refers to probable dementia, which is a substantial issue among the population of interest with well-documented disparities (Alzheimer’s Association, 2019; Matthews et al., 2019) and is associated with limitations in daily functioning (Hall et al., 2011). Probable dementia is captured either by self-report of dementia diagnoses, a score indicating dementia on the AD8 Dementia Screening Interview, or performance on cognitive tests of memory, orientation, and executive function (Kasper et al., 2013). Support characteristics include living arrangement and use of paid help. Living arrangement is based on marital status and household composition. Respondents were categorized as having lived alone, with a spouse only, with a spouse and others, or with others only. We included a measure that reflects whether the person who helped participants with self-care, mobility, household, and selected other tasks (driving, seeing the doctor, taking care of money, and health insurance matters) was paid. This categorization is based on prior work (Freedman & Spillman, 2014; Reckrey et al., 2020).

### Statistical Analysis

We used univariate statistics to describe our total sample, Pearson’s chi-square tests and Student’s *t*-test to determine significant race and gender differences in sociodemographic factors, living arrangement, and health characteristics, as well as receipt of assistance with self-care, mobility, and household tasks. We construct binary logistic regression models to examine whether race and gender differences in receiving help were present after adjusting for demographic (age, education, Medicaid enrollment), health (self-rated health, heart attack, heart disease, diabetes, stroke, dementia), and support (living arrangement, use of paid help) characteristics. Because of the social advantages that have been known to lead to better health and functional outcomes (Bell et al., 2020; Thorpe, Duru, et al., 2015; Warner & Brown, 2011), White males were used as the reference group for each outcome. Odds ratios and corresponding 95% confidence intervals indicate the impact of each predictor and whether it met statistical significance. All analyses were adjusted for sampling weights (DeMatteis et al., 2016a) and used survey estimation commands in Stata 15.0 (StataCorp, 2017) to account for unequal probabilities of selection.

## Results

Table 1 presents descriptive characteristics for the total sample as well as by race and gender. Among those receiving assistance with daily activities, roughly 40% of older adults were between the ages of 65 and 74. Forty-four percent of older adults had obtained more than a high school diploma or GED. Sixteen percent were Medicaid-enrolled. Roughly two thirds of older adults reported either good (33.3%) or fair (31.5%) self-rated health. Health conditions were present in the following order: diabetes (35.6%), heart disease (30.2%), stroke (29.0%), probable dementia (21.2%), and heart attack (13.6%). Approximately 40% lived with a spouse only, followed by living with others only (24.2%), living alone (23.2%), and living with a spouse and others (12.1%). A little more than one quarter of the sample received assistance from a paid helper. Differences by race and gender were observed for most characteristics. Black men were younger, and White women older, than other groups. There were disparities in educational attainment—Black men most often reported not completing high school or obtaining their GED. White women were most often high school graduates. White men most often reported obtaining education beyond high school. Black men and women were more often Medicaid-enrolled compared to White men and women. White women most often reported excellent, very good, or good health. Black women most often reported fair health, and White men most often reported poor self-rated health. White men had the highest rates of heart attack and stroke. Black women had the highest rates of diabetes, and White women had the lowest rates of probable dementia. Regarding living arrangements, white women were more often living alone, White men were more likely to live with a spouse only, Black men were more often living with a spouse and others, and Black women more often lived with others only.

**Table 1.** Distribution of Older Adults Who Report Receiving Assistance With Self-Care, Mobility, or Household Tasks, for the Total Sample and by Race and Gender

Variable	Total	White		Black	
		Men	Women	Men	Women
*N* (%)	1,808 (100%)	437 (32.3%)	796 (53.7%)	158 (4.0%)	417 (10.0%)
Weighted estimates	7,281,166	2,348,238	3,913,455	293,616	725,855
*Demographic characteristics*					
Age, *n* (%)**					
65–74	39.5	42.7	37.3	52.0	36.1
75–84	36.3	37.2	35.4	33.8	39.2
85+	24.2	20.2	27.2	14.1	24.6
Education, *n* (%)***					
Less than high school graduate	22.0	20.0	17.9	46.9	40.9
High school graduate/GED	34.0	30.6	37.8	25.8	27.7
More than high school graduate	44.0	49.4	44.3	27.3	31.4
Medicaid-enrolled, *n* (%)***	16.0	8.7	13.5	40.3	43.3
*Health characteristics*					
Self-rated health**					
Excellent/very good	19.2	19.5	21.5	9.6	9.7
Good	33.3	33.4	34.1	32.4	28.7
Fair	31.5	29.7	29.5	41.9	44.3
Poor	16.0	17.4	14.9	16.1	17.3
Selected health characteristics					
Heart attack***	13.6	20.3	9.3	16.2	13.8
Heart disease	30.2	33.5	25.4	29.0	27.7
Diabetes***	35.6	40.2	29.6	45.5	49.1
Stroke*	29.0	30.6	29.9	28.0	19.4
Probable dementia	21.2	26.6	16.6	26.5.	26.8
*Support characteristics*					
Living arrangement, *n* (%)***					
Lives alone	23.5	13.2	29.8	21.9	24.3
Lives with spouse only	40.2	59.0	34.1	31.5	15.4
Lives with spouse and others	12.1	15.7	10.0	18.6	8.9
Lives with others only	24.2	12.1	26.1	28.0	51.4
Receives paid help	26.3	26.5	25.7	29.6	27.3

*Notes:* GED = General Educational Development. 1,808 community-dwelling Black and White older adults receiving assistance with self-care mobility or household activities (for a health reason). Data are survey weight adjusted.

*p < 0.05, **p < .01, ***p < .001.

Table 2 describes race and gender differences in receipt of assistance by task. First, half of older adults receiving assistance were getting help with self-care tasks. This differed by race and gender—White men were most often receiving help with self-care activities than the other groups. The most common form of assistance was with dressing (41.2%), followed by bathing (25.7%), eating (14.5%), and toileting (9.1%). Significant race and gender differences were only present in the dressing category, with White men most often receiving help, and White women least often receiving help. Second, 44.8% of older adults were getting help with mobility activities. Significant race and gender differences were present. Black women most often received help with mobility activities. Black men least often received help with these activities. Greater than one third of older adults were receiving assistance getting around outside (37.2%), with fewer receiving help getting around inside the house (22.4) and getting in and out of bed (17.4%). Race and gender differences were present for those receiving help getting around outside. Black women were most often receiving help with this task (47.7%), followed by White women (40.8%), White men (29.3%), and Black men (25.2%). Third, nearly 80% of older adults were receiving assistance with household activities. Black women were most often getting help with household activities (88.4%), and White men were the least likely to receive help (69.4%). Race and gender differences were observed for each household task. Black women were more likely to receive help with specific household tasks, except for banking and medication administration. For these activities, Black men were most often receiving assistance. Additionally, compared to other groups, White men were least likely to receive help with banking (along with White women), laundry, meal preparation, and shopping. White women were least likely to receive help with medication administration (23.1%) than other groups.

**Table 2.** Receipt of Assistance, by Race and Gender

Variable	Total	White		Black	
		Men	Women	Men	Women
*N* (%)	1,808 (100%)	437 (32.3%)	796 (53.7%)	158 (4.0%)	417 (10.0%)
Weighted estimates	7,281,166	2,348,238	3,913,455	293,616	725,855
*Types of assistance*					
Any self-care assistance***	51.5	59.7	46.9	48.3	51.3
Bathing	25.7	24.7	24.6	27.6	34.8
Dressing***	41.2	48.6	37.0	39.8	40.6
Eating	14.5	17.7	13.3	13.7	11.6
Toileting	9.1	9.3	9.0	7.9	9.8
Any mobility assistance***	44.8	37.9	48.5	29.8	53.2
Getting around inside	22.4	20.6	23.1	17.1	27.0
Getting around outside***	37.2	29.3	40.8	25.2	47.7
Getting in and out of bed	17.4	17.8	17.1	16.9	17.9
Any household assistance*	79.7	69.4	83.6	87.5	88.4
Banking***	33.4	31.1	31.1	50.4	46.4
Laundry***	39.7	30.8	42.0	49.4	51.7
Meal preparation***	39.8	31.3	42.4	45.3	51.2
Medication administration***	27.5	32.3	23.1	36.7	31.6
Shopping***	61.8	45.3	70.3	55.3	71.9

*Notes:* 1,808 community-dwelling Black and White older adults receiving assistance with self-care mobility or household activities (for a health reason). Data are survey weight adjusted.

*p < 0.05, ***p < .001.

Table 3 presents the logistic regression models describing the association between race and gender and self-care, mobility, and household limitations among those receiving help with daily activities. After controlling for sociodemographic factors, health, and support characteristics, compared to White men, Black men receiving help with daily activities had lower odds of receiving assistance with self-care activities (adjusted odds ratio [AOR] 0.67 [95% confidence interval {CI} 0.47–0.97]). White and Black women had greater odds of receiving assistance with mobility activities (AOR 1.93 [95% CI 1.31–2.83] and AOR 1.72 [95% CI 1.04–2.83], respectively), while Black men had lower odds (AOR 0.56 [95% CI 0.34–0.93]) compared to White men. Relative to White men, White women (AOR 2.20 [95% CI 1.55–3.13]), Black men (AOR 2.69 [95% CI 1.34–5.40]), and Black women (AOR 2.49 [95% 1.43–4.34]) all had higher odds of receiving assistance with household tasks.

**Table 3.** Associations Between Race, Gender, and Receiving Assistance With Self-care, Mobility, and Household Tasks

Characteristics	Self-care			Mobility			Household		
	AOR	95% CI	p	AOR	95% CI	p	AOR	95% CI	p
*Demographic characteristics*									
Race/gender									
White men	Ref			Ref			Ref		
White women	0.82	0.60–1.12	.21	1.93	1.31–2.83	<.001	2.20	1.55–3.13	<.001
Black men	0.67	0.47–0.97	.04	0.56	0.34–0.93	.03	2.69	1.34–5.40	.01
Black women	0.88	0.64–1.23	.12	1.72	1.04–2.83	.03	2.49	1.43–4.34	.01
Age									
65–74	Ref			Ref			Ref		
75–84	0.85	0.61–1.19	.33	0.94	0.68–1.29	.68	1.06	0.74–1.54	.74
85+	1.07	0.73–1.58	.72	1.53	1.08–2.17	.02	1.75	1.09–2.79	.21
Education									
Less than high school graduate	Ref			Ref			Ref		
High school graduate/GED	1.19	0.80–1.76	.39	0.73	0.50–1.07	.10	0.99	0.62–1.58	.98
More than high school graduate	1.08	0.70–1.66	.72	0.90	0.61–1.33	.60	0.84	0.57–1.25	.39
Medicaid-enrolled	1.35	0.93–1.97	.11	1.08	0.78–1.49	.65	1.04	0.63–1.74	.87
*Health characteristics*									
Self-rated health									
Excellent/very good	Ref			Ref			Ref		
Good	0.75	0.51–1.09	.11	1.23	0.82–1.83	.31	1.35	0.87–2.08	.17
Fair	0.73	0.49–1.09	.12	1.67	1.13–2.47	.01	2.20	1.32–3.63	.01
Poor	0.93	0.58–1.49	.76	3.46	2.19–5.47	<.001	3.40	1.74–6.64	<.001
Selected health conditions									
Heart attack	1.11	0.77–1.62	.57	1.06	0.72–1.55	.79	0.77	0.48–1.23	.27
Heart disease	1.12	0.82–1.54	.46	1.00	0.79–1.29	.98	1.20	0.81–1.78	.36
Diabetes	1.47	1.12–1.93	.01	0.92	0.70–1.22	.58	0.71	0.49–1.03	.07
Stroke	0.98	0.70–1.36	.98	1.47	1.12–1.91	.01	1.02	0.62–1.67	.62
Dementia	1.84	1.36–2.48	<.001	2.11	1.55–2.87	<.001	2.97	1.87–4.74	<.001
*Support characteristics*									
Living arrangement									
Lives alone	Ref			Ref			Ref		
Lives with spouse only	4.01	2.70–5.94	<.001	1.92	1.33–2.76	<.001	0.66	0.39–1.13	.13
Lives with spouse and others	3.20	1.99–5.12	<.001	1.73	1.05–2.83	.03	0.69	0.38–1.24	.21
Lives with others only	2.31	1.55–3.43	<.001	2.84	2.00–4.04	<.001	1.19	0.73–1.94	.47
Uses paid help	3.57	2.35–5.40	<.001	3.84	2.72–5.41	<.001	2.10	1.37–3.23	<.001

*Notes:* GED = General Education Development; AOR = adjusted odds ratio; CI = confidence interval. 1,808 community-dwelling Black and White older adults receiving assistance with self-care mobility or household activities (for a health reason). Data are survey weight adjusted.

## Discussion

The objective of the present study was to determine whether, among LTSS recipients, there were race and gender differences in receiving help with daily activities. We find race and gender differences in receiving assistance with self-care, mobility, and household activities after controlling for sociodemographic factors, health, and support characteristics. Findings underscore the importance of examining the roles of both race and gender in receiving assistance with daily tasks and have implications for future research and practice.

White and Black women receiving help with daily activities were more likely to receive assistance with mobility activities compared to White men. These findings are partly supported by prior work that attributes these differences to the presence of chronic health conditions (Vincent et al., 2010). Lack of availability of help might contribute to observed differences—among middle- and older-aged adults in a large epidemiological study, measures associated with lower perceived caregiver availability included being female or White (Roth et al., 2007). Other important considerations include social determinants of health (Thorpe et al., 2008, 2011, 2014; Thorpe, Kelley, et al., 2015). It is possible that environmental factors may be amenable to interventions that better support women requiring assistance with mobility tasks (e.g., proximity to recreational facilities, social support, and transportation), removing the need for a caregiver (Levasseur et al., 2015). Women receiving assistance with mobility activities may be taking advantage of local resources that help them move around inside and outside of their homes.

Relative to White men, Black men receiving help with routine daily activities were less likely to receive assistance with self-care and mobility activities. This finding contrasts prior evidence indicating that Black men have worse physical functioning (Warner & Brown, 2011), which would likely increase the odds of receiving help. Still, some work has shown that Black men have lower odds of disability compared to White men, particularly when living in similar environmental conditions (Thorpe et al., 2014). One possible explanation for this finding requires the consideration of cohort effects. Research has shown that older Black Americans born earlier in the twentieth century had healthier lifestyles than those born later. Specifically, Black people during this time had lower rates of smoking and better nutrition than Whites (Garfinkel, 1984; Popkin et al., 1996). Therefore, it is possible that Black men receiving assistance with daily activities who survived to older age are still a relatively healthier group than older White men. Still, as Black men have lower health care utilization rates and are more likely to delay care (Cheatham et al., 2008; Hammond et al., 2010), it is possible that they also may be less likely to ask for help with self-care and mobility tasks. More information is needed to examine how care preferences and health behaviors of older Black men contribute to the receipt of personal assistance, particularly as it relates to mobility activities.

White and Black women, as well as Black men, were all more than twice as likely to receive assistance with household activities compared to White men. This is supported by prior work that shows that White and Black women, and Black men, are more likely to report limitations with household activities compared to White men (Murtagh & Hubert, 2004; Zsembik et al., 2000). However, recent research reports that measurements of receiving help with household activities do not equivalently measure the same construct for men and women—men are less likely to complete household activities for reasons unrelated to health limitations (Sheehan & Tucker-Drob, 2019). Research has also shown that, in general, older adults more often report receiving assistance with household activities than self-care or mobility tasks (Yorkston et al., 2008), as is the case in the present study. Household activities, which consist of shopping, meal preparation, and other related tasks, typically correspond with receipt of paid assistance from home care providers (Yorkston et al., 2008), and there are gender disparities in the frequency and amount of care provided to men and women. Women receive fewer hours of support from family and friends as well as paid providers, even after considering living arrangements and adjusting for factors such as demographics, disability, and health conditions (Katz et al., 2000). This matter is further complicated by race differences in home care utilization. Research has shown that among older adults using paid services, intensity of services is lower for Blacks compared to Whites (White-Means & Rubin, 2004; Yeboah-Korang et al., 2011). Therefore, despite having higher odds of receiving help with household activities, Black women may still experience consequences because of service gaps. Future research should further examine the implications of race and gender differences in household limitations and work to address inequities that may exist.

Findings from this study have implications for the delivery of paid and unpaid LTSS as well as health service utilization. Our findings indicate that people receiving paid help are more likely to use all forms of assistance. Research is mixed as it relates to finding race differences in the use of paid LTSS among people with disabilities (Fabius et al., 2019; Konetzka & Werner, 2009; Miller et al., 1996). Nevertheless, prior work has demonstrated that, among older adults receiving Medicaid Home and Community-Based Services (HCBS) to assist with daily activities, Blacks have lower Medicaid HCBS expenditures and more often use nonskilled services (e.g., housekeeping), rather than skilled services (e.g., home health), relative to Whites (Fabius et al., 2018). The amount and type of help provided by family and unpaid caregivers as well as paid caregivers likely contribute to the utilization of HCBS. Future studies should examine the distribution of help with self-care, mobility, and household tasks across paid and unpaid caregivers. Second, Black older adults have higher rates of health service utilization, such as hospitalizations and emergency department visits (Fields et al., 2016). While in some cases, older Black adults have greater access to services as a result of financial and functional eligibility, there remain racial disparities in care quality (Konetzka & Werner, 2009), and the confounding of these factors—declining health, increased disability, and poor quality health care—puts Black older adults at further risk for adverse outcomes. The next steps should include evaluations to determine whether older adults receiving assistance with daily activities are getting the appropriate type and amount of help, as evidenced by outcomes such as health service utilization and changes in quality-of-life indicators.

The present study is not without limitations. Due to the cross-sectional nature of our analyses, our results cannot be interpreted as causal. We also cannot speak to the availability of helpers. However, we find that living with others is significantly associated with receiving assistance with self-care and mobility activities—this should be further examined in future studies. Additionally, while we limit our analyses to Black and White older adults, future studies should further examine race and gender differences in assistance with daily activities among other racial and ethnic groups. Indeed, the NHATS collects information on Hispanic ethnicity; however, we were unable to include other relevant factors (e.g., country of origin and nativity, age of migration, duration in the United States) that contribute to differences in disability trends (Garcia et al., 2017). Furthermore, the NHATS combines American Indian, Asian, and Native Hawaiian identities into one category, limiting the ability to draw conclusions about each of these groups. The NHATS also does not ask individuals if they identify as transgender or cisgender. Despite these limitations, this study has several strengths and yields important findings for better understanding race and gender differences in receiving assistance among older Americans. First, as it relates to generalizability, we provide nationally representative estimates, rather than present and interpret findings from a smaller convenience sample. Second, we limit our analyses to those receiving assistance with daily tasks to provide information about community-dwelling older adults who are interacting with LTSS systems.

## Conclusion

Among LTSS recipients, there are race and gender differences in receiving assistance with self-care, mobility, and household activities that exist after adjusting for sociodemographic factors, living arrangements, and health characteristics. Future research should work to better understand factors that contribute to differences, as well as associated consequences that may affect health care utilization and health outcomes. Additionally, the next steps include examining the appropriateness of care, as well as care arrangements for older adults, particularly to understand whether and how the amount and type of help provided by family, friends, or paid helpers differ by race and gender groups.
